# Electrochemical Behavior of Ti6Al4V Alloy Used in Dental Implants Immersed in *Streptococcus gordonii* and *Fusobacterium nucleatum* Solutions

**DOI:** 10.3390/ma13184185

**Published:** 2020-09-21

**Authors:** Myriam A. De la Garza-Ramos, Francisco H. Estupiñan-Lopez, Citlalli Gaona-Tiburcio, Lucía G. Beltrán-Novelo, Patricia Zambrano-Robledo, José Cabral-Miramontes, Facundo Almeraya-Calderón

**Affiliations:** 1 Universidad Autonoma de Nuevo Leon, Facultad de Odontología, Centro de Investigación y Desarrollo de Ciencias de la Salud, Av. Universidad s/n, Ciudad Universitaria, San Nicolás de los Garza, N.L. 66455, Mexico; myriam.garzarm@uanl.edu.mx; 2 Universidad Autonoma de Nuevo Leon, FIME-Centro de Investigación e Innovación en ingeniería Aeronáutica (CIIIA), Av. Universidad s/n, Ciudad Universitaria, San Nicolás de los Garza, N.L. 66455, Mexico; francisco.estupinanlp@uanl.edu.mx (F.H.E.-L.); citlalli.gaonatbr@uanl.edu.mx (C.G.-T.); patricia.zambranor@uanl.edu.mx (P.Z.-R.); jose.cabralmr@uanl.edu.mx (J.C.-M.); 3 Universidad Autónoma de Yucatán, Facultad de Odontología, Calle 60 # 491-A x 57, Centro Histórico, Mérida 97000, Yucatán, Mexico; lgbn17@gmail.com

**Keywords:** dental implants, MeSH, *Streptococcus gordonii*, *Fusobacterium nucleatum*, corrosion, titanium, stainless steel

## Abstract

The titanium alloy, Ti6Al4V, is used in dentistry for dental implants because of its excellent resistance to corrosion and its high biocompatibility. However, periimplantitis is considered the main reason for treatment failure. The Ti6Al4V alloy was used to study the corrosion behavior for dental implant applications, using an experimental arrangement of three electrodes with the bacteria *Streptococcus gordonii* and *Fusobacterium nucleatum*, in addition to Ringer’s lactate as electrolytes, at 37 °C and a pH of 5.6. Their electrochemical behavior was studied by open circuit potential (OCP) and cyclic potentiodynamic polarization (CPP) according to ASTM G3-14 and ASTM G61-11, respectively. Scanning electron microscopy (SEM) was employed to determine the morphology of the alloy studied. An experimental model, in situ, was established with the bacteria present in an oral environment to understand the electrochemical behavior of the alloy used in dental implants. The greatest corrosion in Ti6Al4V alloy was produced by the medium that contained the bacterium *Streptococcus gordonii*, which is considered a primary colonizer. In addition, the Ti6Al4V alloy presented uniform corrosion in the three solutions at the different exposure times showing a negative hysteresis in CPP.

## 1. Introduction

The pure titanium and titanium alloys are known for their use in dental practice owing to their good corrosion resistance, biocompatibility, and biofunctionality in the human body [1,2,3,4,5]. The use of titanium dental implants has revolutionized oral implantology. In the USA, about 300,000 patients a year currently receive dental implants [6,7].

Periodontal disease is one of the main causes of loss of dental support tissue, and therefore the reason for dental loss. It is known that its origin is mainly bacterial [8,9]. There are many stages of periodontal disease, but in the face of imminent progression, treatment such as scaling and root planing, periodontal surgery, and tooth extraction, when the disease is very severe, can be recommended; in severe cases, mastication can be difficult.

One of the most frequent, currently used treatments is the replacement of lost teeth using dental implants [10,11,12]. The success of dental implants depends on an adequate osseointegration process that consists of connecting bone tissue with the dental implant, providing support and anchoring; this procedure takes three to five months to complete [13,14]. However, implants can suffer periimplantitis [15]. Periimplantitis is an inflammatory disease of the soft tissue surrounding the dental implant. It is a pathologic process that is considered a failure in the medium-term of rehabilitation of lost teeth for periodontal reasons, and it is also considered a new health problem for those who believe that it is a solution for the pathological tooth loss [16,17]. Implants are susceptible to colonization by bacteria in the oral cavity [18], which has a large bacterial population that covers all of its surfaces. This “biofilm” is a group of microorganisms that adhere to the mucosa, tissue, teeth, and any material placed in the mouth [19,20]. The bacteria that form the supra or subgingival oral biofilm possess different characteristics that naturally do not produce negative effects; however, if the population of microorganisms increases, this can be detrimental and facilitate the appearance of diseases such as periimplantitis [16]. The bacteria *Fusobacterium nucleatum* and *Streptococcus gordonii* [21,22] have been found to be some of the most important related to periimplantitis in patients, mainly in smokers [23,24]. Despite demonstrating excellent performance with a clinical failure rate lower than 10%, the main cause of dental implant loss is periimplantitis [25].

In the manufacture of implants, among these, dental and other biomedical devices, titanium is commonly used because of its properties, such as its high biocompatibility, good osseointegration, high resistance\weight ratio, its excellent resistance to corrosion [14,26], and excellent corrosion resistance by the formation of a passive film consisting mainly of amorphous titanium dioxide (TiO_2_) [27]. Studies have been performed regarding the electrochemical behavior of titanium implants with artificial saliva at different pH values [28,29]. These have demonstrated that there is a greater concentration of free titanium ions at low pH and a greater density of corrosion flow as also shown in other studies under diverse conditions [27,30,31]. The titanium oxidation reaction is as follows.

Ti → Ti (IV) + 4e^−^ (1)

O_2_ + 4H^+^ + 4e^−^ → 2H_2_O (2)

The objective of this research was to evaluate the corrosion behavior of the Ti6Al4V alloy in the presence of bacterial solutions of *Streptococcus gordonii*, *Fusobacterium nucleatum* + *Streptococcus gordonii*, and a reference media at 37 °C and a pH of 5.6. Scanning electron microscopy (SEM) was employed to determine the morphology of the alloys studied. Their electrochemical behavior was studied by open circuit potential (OCP) and cyclic potentiodynamic polarization (CPP).

## 2. Materials and Methods

### 2.1. Material

The material used was an alloy of Ti6Al4V, which was obtained from 12.7 mm diameter bars cut into cylinders 25 mm high. The samples were prepared by metallography up to grade 800 silicon carbide sandpaper, ultrasonic cleaning for 15 min in ethanol, and drying with pressurized air. The chemical composition of the Ti6Al4V alloy was obtained by X-ray fluorescence (Olympus DELTA XRF, Webter, TX, USA) [32].

The test solutions were prepared with tryptic casein broth (TSB) medium in a glass flask with the bacteria *Streptococcus gordonii* and *Fusobacterium nucleatum*, which were activated at 37 °C for 5 h. In one solution, only *Streptococcus gordonii* bacteria were used, while the other was a mixture of both bacteria [33], where inoculation was in a 1:1 ratio in a new sterile TSB medium at a concentration of 1 × 10^6^ CFU/mL with a McFarland turbidity of 0.5 in a final volume of 1 mL with incubation at 37 °C for 12 h. Ringer’s lactate was used as a control solution with a chemical composition of NaCl 425 mg, KCl 15 mg, CaCl_2_ 10 mg, 2.58 mL of sodium lactate 60% in 500 mL of distilled water [34].

Every 24 h, one-quarter of the TSB medium was taken from the flask and replaced with new sterile TSB to provide nutrients to the bacterial population to keep it in a potential state of growth due to the need for nutrients and to constantly conserve biomass, since in response to changes in environmental conditions or signals, a bacterial cell can activate or deactivate the production of specific protein molecules. This is known as gene regulation; if this is not done, it can influence the production or overproduction of certain enzyme proteins and can modify the pattern of corrosive components that affect the experiment [34]. The pH values of the bacteria were, Table 1:

### 2.2. Electrochemical Techniques

A conventional three-electrode cell configuration was used for the electrochemical studies, which consisted of a working electrode (titanium alloy (Ti6Al4V)), a saturated calomel electrode (SCE), and a platinum mesh, which were used as a reference and counter electrode, respectively. Electrochemical measurements were carried out using an electrochemical interface and a Solartron 1287 phase gain. Corrosion experiments were performed by immersion of the titanium alloy (Ti6Al4V) specimens, with an exposed surface area of 1.0 cm^2^, in presence of bacterial solutions of *Streptococcus gordonii*, *Fusobacterium nucleatum* + *Streptococcus gordonii*, and a reference media, at a temperature of 37 °C. The monitoring times were 0, 48, and 96 h. The electrochemical techniques of open circuit potential (OCP) and cyclic potentiodynamic polarization (CPP) were used to determine the corrosion kinetics of Ti6Al4V alloy. The open circuit potential (OCP) was recorded at 60 min and 1 data/s, according to ASTM G3-14 standard [35] and the CPP was recorded at a sweep rate of 0.06 V/min; a potential scan range was applied between −0.20 and 1.5 V vs. SCE, from the corrosion potential (E_corr_), using a complete polarization cycle, according to ASTM G61-11 standard [36]. In CPP curves, analysis of the cathodic and anodic branches and the hysteresis curve can yield information about the corrosion process in the system, as well as the corrosion rates.

Tafel extrapolation of potentiodynamic polarization curves is used to determine the corrosion current density, *i*_corr_ (mA·cm^−2^) from which it is possible to determine the corrosion rate [37,38,39].

The corrosion kinetic behavior using potentiodynamic polarization can be observed through cathodic and anodic reactions in polarization curves. Corrosion rate in terms of penetration (mm/s) is one of the main parameters obtained by potentiodynamic polarization curves, according to Faraday’s law (Equation (3)) [40,41,42].

(3) $$Corrosion\;rate=K_{1}\frac{\left(I_{corr}\right)}{\rho }\;E.W$$

where *E.W* = equivalent weight, *I_corr_* = current density in μA/cm^2^, and ρ = density in g/cm^3^.

### 2.3. Microstructural Characterization

Analysis of surface morphology and composition was done using a scanning electron microscope (SEM, Jeol JSM 6510LV, Tokyo, Japan). After finalizing electrochemical testing, the samples were analyzed to determine the corrosion products at magnifications of 100, 500, and 1000X, operating at a voltage of 20 kV and a working distance (WD) of 10 mm. The chemical composition of the surface was obtained by energy dispersive X-ray spectroscopy (EDS).

## 3. Results

### 3.1. Chemical Composition

The chemical composition of the material under study obtained by X-ray fluorescence is presented in Table 2.

### 3.2. Open Circuit Potential (OCP)

**Figure 1:**
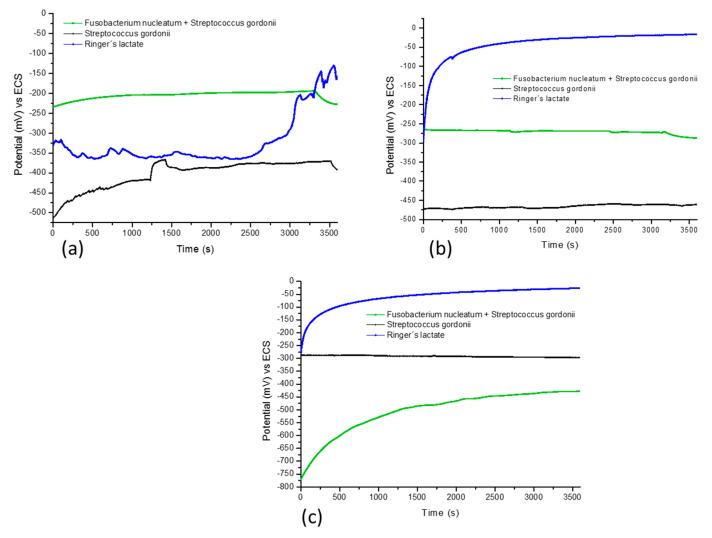
Open circuit potentials of titanium alloy (Ti6Al4V) at 0 (**a**), 48 (**b**), and 96 (**c**) hours of exposure in: *Fusobacterium nucleatum* + *Streptococcus gordonii*; *Streptococcus gordonii*; and Ringer’s lactate solution at 37 °C.

The behavior of the open circuit potential of the Ti6Al4V alloy revealed general information regarding the interaction between the metal surface and the electrolyte. At exposure times of 0, 48, and 96 h, shown in Figure 1, recorded for one hour at 37 °C, the different media (bacteria) *Fusobacterium nucleatum* + *Streptococcus gordonii*, *Streptococcus gordonii*, and Ringer’s lactate were observed (Table 3).

**Figure 2:**
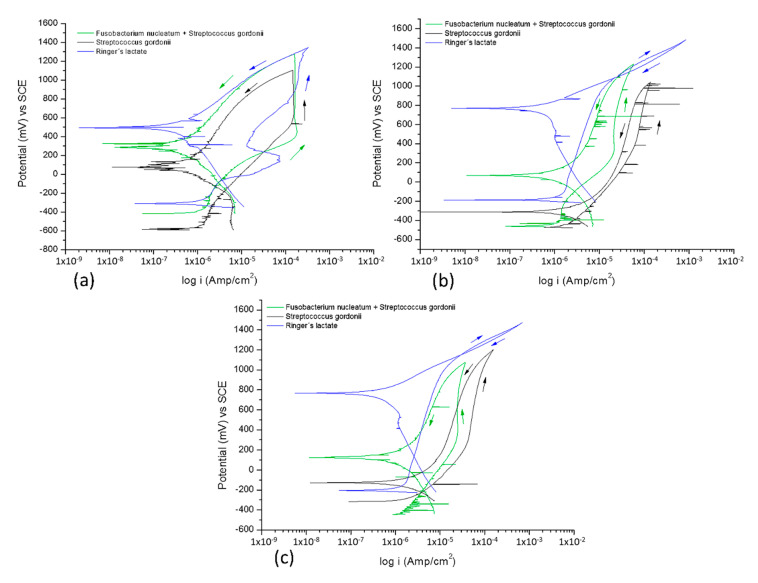
Cyclic potentiodynamic polarization of the Ti6Al4V alloy a 0 (**a**), 48 (**b**), and 96 (**c**) hours of exposure in: *Fusobacterium nucleatum + Streptococcus gordonii*; *Streptococcus gordonii*; and Ringer’s lactate at 37 °C.

Ti6Al4V alloy in the bacteria *Fusobacterium nucleatum* + *Streptococcus gordonii* initiated active corrosion potentials, and at exposure times of 0 and 48 h, the material tended to present stable corrosion potentials (Figure 2a,b). When the exposure time of 96 h passed, these potentials tended to be more noble (Figure 2c). The mean values of OCP were −423 and −383 mV, respectively.

In the presence of the bacterium *Streptococcus gordonii*, the study alloy presented a trend toward noble potentials at the start of exposure, beginning with action potentials of −510 mV (see Figure 2a,b). At 96 h, the OCP of the alloy started with action potentials of −287 mV and tended to stabilize at potentials of −297 mV as the exposure time increased, showing that the oxidation current of the alloy was reduced and formed a passive film.

The behavior of the Ti6Al4V alloy in the presence of Ringer’s lactate is shown in Figure 2a–c, at 0 h, begins with potentials of −320 mV with fluctuations, to end at noble potentials (−120 mV), while at 48 and 96 h, the potentials have a trend toward positive values. At the end of 96 h, the titanium alloy presented more negative potentials in the three electrolytes due to the greater stability of the passive layer on the surface.

### 3.3. Cyclic Potentiodynamic Polarization (CPP)

The cyclic potentiodynamic polarization curves organized by the electrolyte at different immersion times are shown in Figure 2. Figure 2a corresponds to the bacterial solution *Fusobacterium nucleatum + Streptococcus gordonii*, Figure 2b to the bacterium *Streptococcus gordonii*, and Figure 2c to Ringer’s lactate. The corresponding corrosion parameters are given in Table 4 and obtained from the cyclic potentiodynamic curves that determine the susceptibility to pitting corrosion. Ti6Al4V alloy does not present a localized corrosion behavior.

Pitting corrosion resistance, R_pit_, is determined by the absolute value of the difference between the E_corr_ and E_pit_ [37]. However, the titanium alloys did not show a positive hysteresis, therefore it does not have the potential for pitting (E_pit_).

In the cyclic potentiodynamic polarization curves, a cathodic and anodic reaction of the Ti6Al4V alloy is observed in the bacteria solution *Fusobacterium nucleatum + Streptococcus gordonii*, which presents a uniform corrosion behavior at different exposure times typical of a negative hysteresis. The titanium alloy tends to present a slight increase in its *i_corr_* when exposure time increases. At 0 and 48 h of exposure, activation of the anodic branch curves is observed, followed by pseudo-passivation (approximately −300 mV), where the density of the corrosion current does not significantly increase when the corrosion potential increases. This behavior is also observed in results from the literature [38]. Later, trans-passivation occurs with an increase in current; however, the evaluated titanium alloy at 96 h did not present a pseudo-passivation behavior until 300 mV.

The Ti6Al4V alloy in Ringer’s lactate at 0 h has an activation behavior in the anodic branch, pseudo-passivation, trans-passivation, secondary passivation, and again a rupture of the passive film, showing negative hysteresis in the return of the potential sweep. At 48 and 96 h, the hysteresis loop is very small, in other words, the anodic branch of the reverse sweep potential returns in the same direction, indicating that there is no tendency toward localized corrosion and its behavior is activation with increases in low corrosion current densities. The titanium alloy behavior shows a localized corrosion process (positive hysteresis), independent of the immersion time in the bacteria.

**Figure 3:**
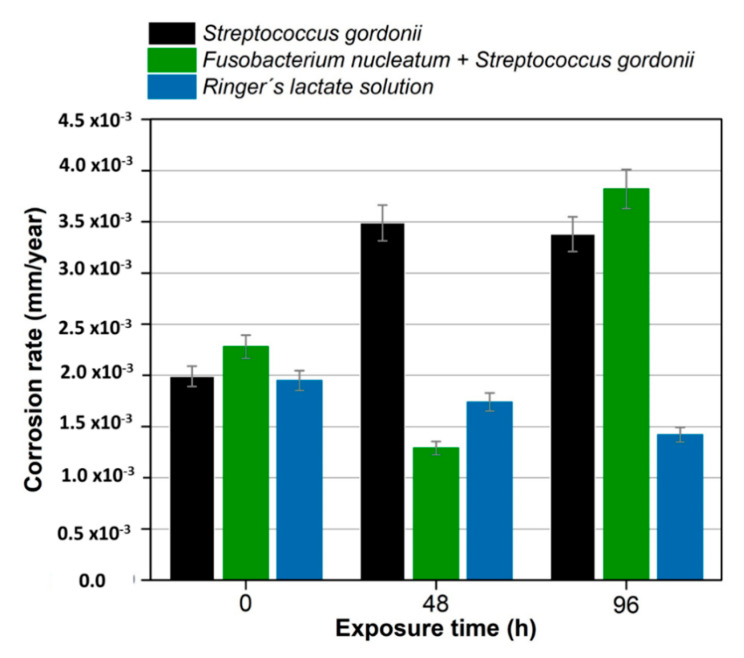
Corrosion rate of the Ti6Al4V alloy at 0, 48, and 96 h of exposure to *Streptococcus gordonii*, *Fusobacterium nucleatum* + *Streptococcus gordonii*, and Ringer’s lactate at 37 °C.

Figure 3 shows the behavior of the corrosion rate as a function of the exposure time for the titanium alloy in the presence of the different bacteria (*Fusobacterium nucleatum + Streptococcus gordonii* and *Streptococcus gordonii*) under study. Corrosion rate values for bacteria are in the order 10^−3^ mm/year, at different exposure times.

### 3.4. SEM Microstructural Analysis

**Figure 4:**
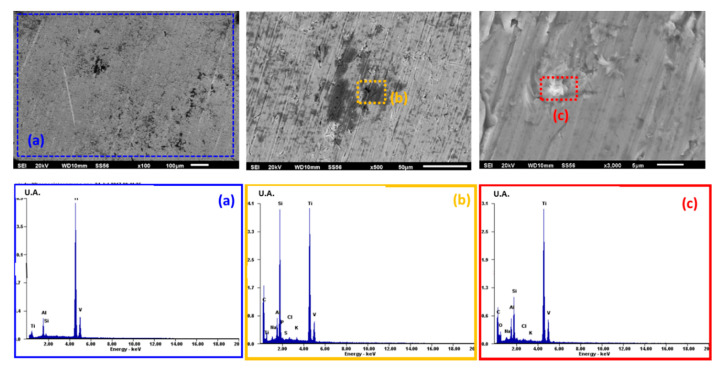
SEM surface morphology micrographs and EDS spectrum of Ti6Al4V in *Streptococcus gordonii:* (**a**) 100X, (**b**) 500X, and (**c**) 2000X.

**Figure 5:**
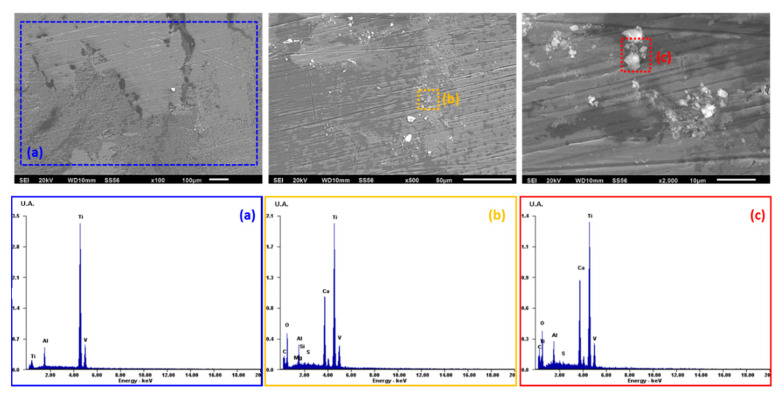
SEM surface morphology micrographs and EDS spectrum of Ti6Al4V in *Fusobacterium nucleatum + Streptococcus gordonii:* (**a**) 100X, (**b**) 500X, and (**c**) 2000X.

**Figure 6:**
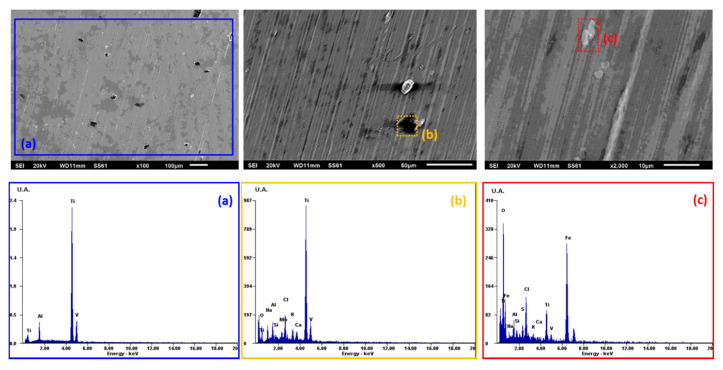
SEM surface morphology micrographs and EDS spectrum of Ti6Al4V in Ringer’s lactate: (**a**) 100X, (**b**) 500X, and (**c**) 2000X.

The morphology micrographs of the study alloys and the elements present on the surface after the electrochemical experiments performed at 96 h were analyzed by scanning electron microscopy (SEM) and energy-dispersive X-ray spectroscopy (EDS). Micrographs obtained by SEM (100, 500, and 2000X) and the analysis of the Ti6Al4V alloy by EDS after the electrochemical test of potentiodynamic polarization at 96 h are shown in Figure 4, Figure 5 and Figure 6.

Figure 4, which corresponds to the *Streptococcus gordonii* solution, shows the morphology without corrosion products on the surface. In the EDS spectrum, characteristic elements of the Ti6Al4V alloy are seen (titanium, aluminum, vanadium, silicon), in addition to the presence of rich deposits of silicon and carbon, as well as traces of sodium, potassium, sulfur, phosphorus, and chloride.

Micrograph and EDS analyses of the Ti6Al4V alloy in the *Fusobacterium nucleatum + Streptococcus gordonii* bacterial solution are shown in Figure 5. Calcium-rich deposits are seen, in addition to the characteristic elements of the alloy, such as titanium, aluminum, and vanadium (EDS analysis, blue square a; 100X). A uniform morphology without localized corrosion is observed.

Micrograph and EDS analysis of the Ti6Al4V alloy in Ringer’s lactate is shown in Figure 6. The metal surface does not present deposits of species with an organic nature, in contrast to solutions that contain bacteria. The elements present are characteristic of the metal base and Ringer’s lactate. The 100X micrograph shows some areas with pitting corrosion.

## 4. Discussion

The presence of metallic elements in the oral cavity may lead to the formation of electro-galvanic cells and, as a result, may induce corrosion [42]. The biofilm formation process leads to a change of oral cavity parameters, such as electrolytic concentration, pH, or oxygen levels [43].

Cyclic potentiodynamic polarization method is a highly useful method for determining the susceptibility of a metal or alloy to pitting [17].

Ti6Al4V alloy, in the presence of the study electrolytes (*Streptococcus gordonii*, *Fusobacterium nucleatum* + *Streptococcus gordonii*), allows the formation of a passive TiO_2_ (titanium dioxide) film on the electrolyte/metal interface [26]. This slows the corrosion rate since it acts as a barrier to metal dissolution. A change in the electrolyte composition (for example, pH and bacteria life) can reduce the corrosion rate in the implant as mentioned by Bhola, et al. [4]. The Ti6Al4V alloy at 48 and 96 h presents stable behavior of the passive film, demonstrating that the electrochemical reactions on the surface of the alloy are in balance [44,45]. This is consistent with Grosgogeat et al. [46], who studied the electrochemical behavior of titanium in near-real clinical conditions and concluded that it is essential to have oxidated layers that are characteristic of a pseudo-stationary state. In addition, Revie [47] and Fraker [48] demonstrated that the passiveness of a titanium oxide layer depends mainly on the duration of immersion and represents a process of aging. These results are consistent with that found in previous studies of the formation of a passive film on a titanium surface [49,50]. At the end of 96 h of exposure, the titanium alloy presents more noble potentials (−285 mV) in the solution of the bacteria *Fusobacterium nucleatum + Streptococcus gordonii* in comparison to that recorded with the bacterium *Streptococcus gordonii* (−550 mV); this indicates a greater tendency towards corrosion. In general, it is accepted that reducing sulfate-reducing bacteria (SRB) are the major causing corrosion processes [51]. However, there are some representatives of other anaerobic pathogens of the Gram-negative anaerobic family, such as *Fusobacterium nucleatum* and *Prevotella melaninogenica*, which produce butyric acid, carbon dioxide, and hydrogen during the enzymatic degradation of saccharides, and this process can increase the corrosion rate of the titanium alloy used in this work [52]. In addition, their metabolic activity in combination with organic compounds can lead to the formation of aggressive corrosion products, e.g., organic acids [53].

The *Streptococcus gordonii* solution presents more noble potentials at 0 and 48 h; due to the passive TiO_2_ film, it is more stable and does not allow the bacteria to react immediately even though it is an early colonizer. In Ringer’s lactate, Ti6Al4V showed more noble potentials at different exposure times than the bacteria solutions, obtaining values of −80 mV (Ti6Al4V) at 96 h of exposure. Some authors also found a poor corrosion resistance of Ti alloy (Ti-13Nb-13Zr and Ti-6Al-4V) in Ringer’s solution at low pH [54]. The increase in OCP in Ringer’s lactate solution from −301 mV to −67 mV in a period of immersion of 96 h suggests the growth of the passive oxide layer on the metal surface in this solution [55].

The physiology of the microorganisms is calculated at 96 h. The bacteria can decline and change their corrosive capacity due to the metabolisms of both bacteria, since until now, it has been assumed that all cells within a bacterial population are identical. However, in many cases, genetically identical bacteria within a population can show variations in their levels of gene expression [56]. The amount of growth from a specific point during the cell cycle, such as the start of replication or cell division, can be given by Equation (4) [17]:

(4) $$V_{x}=\frac{N_{dt}\;dl}{N_{dl}\;dt}$$

Ti6Al4V alloy in the *Fusobacterium nucleatum + Streptococcus gordonii* bacteria solution presents uniform corrosion at the different exposure times, showing a negative hysteresis. *Streptococcus gordonii* bacteria on the Ti6Al4V alloy showed an activation behavior on the anodic branch of the polarization curves and after half a cycle of polarization, there was a negative hysteresis, characteristic of a generalized corrosion process [52,57]. In this case, there is no secondary passivation behavior, unlike the solution containing the mixture of *Fusobacterium nucleatum + Streptococcus gordonii* since *S. gordonii* grows slowly in the presence of *L-lactate*. This suggests that *S. gordonii* has the ability to metabolize L-lactate, especially in a method that simulates an abscess, and this is useful for measuring infections with *Aggregatibacter actinomycetemcomitan*, because it lacks the ability to metabolize L-lactate in monoculture models. These data suggest that L-lactate produced by *S. gordonii*, and also potentially by host lactate dehydrogenase in subgingival lesions, may influence the growth of this pathogen in the human oral cavity [58,59].

The OCPs found suggest the formation of a passive TiO_2_ film, according to the Pourbaix diagram for the titanium (Ti)-water system [38]. These oxides can be anatase and rutile and their formation depends on the pH of the solution [60], which can be formed in the corresponding pH range to an oral environment.

The titanium alloys used in dental implants and evaluated in Afnor artificial saliva have a passivation behavior in the anodic branch [38], similar to that found in Ringer’s lactate tests. The TiO_2_ film possesses a high corrosion resistance in various test solutions, such as artificial saliva, Ringer’s solution, 0.9% NaCl solution, or physiological saline solution [61,62]. The comparison of the electrochemical behavior identified by the potentiodynamic polarization tests is notable in the anodic branch when artificial solutions or bacteria are used. The higher corrosion rate in Ti6Al4V alloy is produced by the medium containing *Streptococcus gordonii* and is due to the fact that it is a primary colonizer [63]; that is, it is the one that adheres first to the metal surface.

SEM micrographs were taken after cyclic potentiodynamic polarization tests to analyze the morphology and elements present by EDS. The Ti6Al4V alloy in *Streptococcus gordonii* has deposits rich in silicon and carbon detected by EDS and derived from the microbial metabolism of casein, which is the protein of the culture medium used (TSB: casein + bi-distilled water), and from the sodium (Na) and potassium (K) of the water used, while traces of sulfur are associated with the amino acids (methionine and tryptophan) present in the solution due to organic decomposition or bacterial metabolism. Exposure to the bacteria *Fusobacterium nucleatum + Streptococcus gordonii* causes a uniform corrosion morphology with calcium-rich deposits derived from cysteine, which in turn comes from the casein present in the bacterial system. The phosphorus comes from the organic decomposition of the secondary metabolism of casein in the culture medium added with Hemin [38].

High concentrations of carbon are produced by the presence of microorganisms that lodge in pits once localized corrosion damage is generated. The semi-spherical morphology of the pits is due to the morphology of *Streptococcus gordonii* [17,64]. The presence of phosphorus is due to the generation of energy derived from ATP (adenosine triphosphate) [38]. The *Fusobacterium nucleatum + Streptococcus gordonii* solution causes a greater density and size of the pits, with spherical morphology, while non-alloy elements are identified as a product of the microbial mechanisms described.

Gram-negative microbial species, such as *Porphyromonas gingivalis*, *Eikenella corrodens*, *Fusobacterium nucleatum*, and *Parvimonas micra*, have been associated with inflammation and bone loss around osseointegrated implants. Therefore, we have proposed to use the combination of *Streptococcus gordonii*, which is a Gram-positive primary colonizer with *Fusobacterium nucleatum*, both of which can increase the corrosive power on a Ti surface [38,65,66].

Recent research in the field of tissue engineering has investigated new nanotechnologies in order to offer clinicians and patients a better management of the field of regenerative medicine. Having a dental implant, which is also in contact with the oral cavity, does not favor the formation of a biofilm and remains unscathed, which can only be an advantage. Indeed, at this point and only at this point could it create the ideal conditions for re-osteointegration [67].

## 5. Conclusions

- This work presents a study on the corrosion behavior of Ti6Al4V alloy exposed with bacteria (simulating an oral environment). Their electrochemical behavior was studied by open circuit potential and cyclic potentiodynamic polarization.
- After 96 h of exposure, the Ti6Al4V alloy had the most active potentials in the three mediums because of the greater stability of the passive layer on the surface due to the formation of a passive film in the electrolyte-metal interface.
- Cyclic potentiodynamic polarization results indicated that the greater corrosion rate in Ti6Al4V alloy at 96 h of exposure is produced by the medium that contains the bacteria *Streptococcus gordonii* (3.38 × 10^−3^ mm/yr for Ti6Al4V); this is attributed to the fact that it is a primary colonizer.
- The Ti6Al4V alloy in the three solutions (*Fusobacterium nucleatum + Streptococcus gordonii*, *Streptococcus gordonii*, and Ringer’s lactate) presents a uniform corrosion behavior at different exposure times.
- SEM observations indicated that morphology does not present localized corrosion in the Ti6Al4V alloy in the three solutions *Fusobacterium nucleatum + Streptococcus gordonii*, *Streptococcus gordonii*, and Ringer’s lactate. In the *Fusobacterium nucleatum + Streptococcus gordonii*, *Streptococcus gordonii* solutions, deposits rich in silicon and carbon, derived from the microbial metabolism of casein, were identified.
- Given the enormous medical importance of these types of dental implants (the titanium alloys), and in order to obtain a better understanding of their corrosion behavior, it is recognized that the use of powerful techniques such as Electrochemical Impedance Spectroscopy, EIS, would be of great benefit.

## Figures and Tables

**Table 1:** pH measurements of solutions.

Exposition (h)	pH		
	*Fusobacterium nucleatum + Streptococcus gordonii*	*Streptococcus gordonii*	Ringer’s Lactate Solution
0	5.6	5.7	7
48	6.7	6.5	7
96	6.9	6.8	7

**Table 2:** Chemical composition of Ti6Al4V alloy (wt.%).

Alloys	Elements											
	Cr	Ni	Si	Mn	S	C	N	Al	O	V	Ti	Fe
Ti6Al4V	-	-	0.44	-	-	0.04	0.015	6.1	0.18	4	Bal	0.183

**Table 3:** Open circuit potential values of Ti6Al4V alloy at 37 °C.

		Open Circuit Potential (mV) vs. Saturated Calomel Electrode (SCE)					
		*Fusobacterium nucleatum + Streptococcus gordonii*		*Streptococcus gordonii*		Ringer’s Lactate Solution	
Exposure Time (h)	Material	Start	End	Start	End	Start	End
0	Ti6Al4V	−234 ±12	−227 ±11	−510 ±26	−392 ±20	−330 ±17	−157 ±8
48	Ti6Al4V	−264 ±13	−287 ±14	−472 ±24	−461 ±23	−294 ±15	−17 ±0.85
96	Ti6Al4V	−772 ±39	−427 ±21	−287 ±14	−297 ±15	−280 ±14	−27 ±1.35

**Table 4:** Electrochemical parameters using cyclic potentiodynamic polarization of the Ti6Al4V alloy.

Exposure Time (h)	Material	*Fusobacterium gucleatum* + *Streptococcus gordonii*			*Streptococcus gordonii*					Ringer’s Lactate Solution			
		*E_corr_* (mV)	*i_corr_* (nA/cm^2^)	*Corr.Rate* (mm/Year)	*i_pass_* (A/cm^2^)	*E_corr_* (mV)	*i_corr_* (nA/cm^2^)	*Corr. Rate* (mm/Year)	*i_pass_* (A/cm^2^)	*E_corr_* (mV)	*i_corr_* (nA/cm^2^)	*Corr. Rate* (mm/Year)	*i_pass_* (A/cm^2^)
0	Ti6Al4V	−417 ±21	140 ±7	2.28 × 10^−3^ ±1 × 10^−4^	1.70 × 10^−6^ ±9 × 10^−8^	−586 ±29	122 ±6	1.99 × 10^−3^ ±10 × 10^−5^	1.52 × 10^−6^ ±8 × 10^−8^	−309 ±15	120 ±4	1.95 × 10^−3^ ±10 × 10^−5^	2.05 × 10^−6^ ±1 × 10^−7^
48	Ti6Al4V	−461 ±23	79 ±4	1.29 × 10^−3^ ±6 × 10^−5^	1.37 × 10^−6^ ±7 × 10^−8^	−473 ±24	214 ±11	3.49 × 10^−6^ ±2 × 10^−4^	4.46 × 10^−6^ ±2 × 10^−7^	−187 ±9	107 ±3	1.74 × 10^−3^ ±9 × 10^−5^	1.71 × 10^−6^ ±9 × 10^−8^
